# Linear accelerator–based stereotactic arrhythmia radioablation for paroxysmal atrial fibrillation in elderly patients: results from long-term follow-up

**DOI:** 10.3389/fcvm.2026.1881828

**Published:** 2026-07-20

**Authors:** Antonio Di Monaco, Imma Romanazzi, Ilaria Bonaparte, Alessia Surgo, Alba Fiorentino, Massimo Grimaldi

**Affiliations:** 1 Department of Cardiology, General Regional Hospital “F. Miulli”, Acquaviva Delle Fonti, Bari, Italy; 2 Department of Cardiology, Ospedale “San Paolo”, Bari, Italy; 3 Department of Radiation Oncology, General Regional Hospital “F. Miulli”, Acquaviva Delle Fonti, Bari, Italy; 4 Department of Medicine, LUM University, Casamassima, Italy

**Keywords:** atrial fibrillation, elderly, long-term follow up, pulmonary vein isolation, stereotactic arrhythmia radioablation

## Abstract

Stereotactic arrhythmia radioablation (STAR) is an innovative therapeutic approach for cardiac arrhythmias, particularly ventricular tachycardia. Regarding atrial fibrillation (AF), the only study evaluating the safety and feasibility of STAR (photon linear accelerator-based) in elderly patients was published in 2,023. In this brief communication, we reported the long-term follow-up of the enrolled patients. The current data demonstrate the feasibility of STAR in elderly patients with paroxysmal AF and its potential to improve quality of life. The treatment remains experimental, and more comprehensive safety data are necessary; furthermore, its efficacy will require further evaluation in a comparative study vs. catheter ablation.

## Introduction

Stereotactic arrhythmia radioablation (STAR) is an innovative therapeutic approach for cardiac arrhythmias, particularly ventricular tachycardia (1). Concerning atrial fibrillation (AF), the only study evaluating the safety and feasibility of STAR (linear accelerator-based) in elderly patients was published in 2023 (2). In this brief communication, a pre-specified long-term follow-up (FU) of the patients enrolled was reported.

## Methods

Patients aged 70 years or older with symptomatic AF and ineffective or unusable antiarrhythmic therapy were enrolled (2, 3). All clinical characteristics are reported in the Table 1. All patients underwent 4D cardiac computed tomography simulation imaging. Several Organs at Risk (OaRs) were contoured, paying particular attention to the oesophagus and main bronchus, for which a planning risk volume (PRV) was created. The clinical target volume (CTV) was defined around the pulmonary veins (PVs). From CTV, an internal target volume (ITV) was created to account for heart and respiratory motion. Finally, the planning target volume (PTV) was defined by adding 0–3 mm to the ITV, except in the overlap area with OaRs/PRV, where the PTV was cropped. We performed a “simultaneous integrated protection” (SIP) dose for the oesophagus and bronchus; it was applied at the interface between PVs and critical structures to meet the dose constraint and reduce the risk of side effects (2). STAR was performed with a total dose of 25 Gy (single fraction), delivered with high precision to the target and organs at risk, in a very short treatment time (3 min beam-on time) (2–6).

**Table 1:** Clinical characteristics of the 20 study patients.

Patients' characteristic	
Male sex, *n* (%)	8 (40)
Female sex, *n* (%)	12 (60)
Mean age (years)	77 ± 6
CHA2DS2-VASc, *n* (%)	
0	0 (0)
1	1 (5)
2	1 (5)
3	12 (60)
4	6 (30)
EHRA SCORE, *n* (%)	
2 A	–
2 B	–
3	16 (80)
4	4 (20)
Diabetes mellitus, *n* (%)	1 (5)
Hypertension, *n* (%)	16 (80)
Family history of coronary artery disease, *n* (%)	15 (75)
Hypercholesterolemia, *n* (%)	11 (55)
Hypertriglyceridemia, *n* (%)	9 (45)
Active smoking, *n* (%)	6 (30)
Body mass index (Kg/m^2)^	26 ± 3
Heart failure (EF < 35%), *n* (%)	0 (0)
Coronary artery disease, *n* (%)	1 (5)
Previous ischemic stroke, *n* (%)	1 (5)
Transient ischemic attack, *n* (%)	1 (5)
Chronic renal failure, *n* (%)	7 (33)
Dysthyroidysm, *n* (%)	7 (35)
Chronic lung disease, *n* (%)	4 (20)
Medical therapy, *n* (%)	
Beta blockers, *n* (%)	15 (75)
Flecainide, *n* (%)	7 (35)
Propafenone, *n* (%)	1 (5)
Amiodarone, *n* (%)	5 (25)
Sotalol, *n* (%)	2 (10)
Direct oral anticoagulant, *n* (%)	20 (100)

## Results

Since May 2021, 20 patients have been enrolled, and 18 have undergone STAR (2). Longitudinal FU beyond the 12-month post-STAR timepoint specified in the study protocol was maintained for most patients through clinical evaluations, ECG, echocardiography, and Holter monitoring, conducted at least once annually. For patients unable to attend on-site visits, FU was conducted via structured telephone interviews to assess adverse events and AF recurrence. All the safety and efficacy endpoint data are reported in the Table 2.

**Table 2:** Safety and efficacy endpoint data.

Safety endpoint	Efficacy endpoint
Events that are likely related to STAR: – 1 patient developed torsade de pointes one hour after treatment and was successfully treated with electrical cardioversion. – Mild cases of oesophagitis occurred soon after STAR treatment and resolved fully. No patient has reported any further episodes of epigastric pain during the FU period so far. – At 12-month FU, 8 patients (44%) developed an asymptomatic, mild pericardial effusion (≤2 mm); at long term FU, only one patient still has a minimal, asymptomatic effusion, while the others have fully resolved. Events that are not related to STAR: – A patient with known calcific degeneration of the aortic valve (mild stenosis before STAR treatment) developed progressive valve degeneration with severe stenosis documented. – Two patients underwent pacemaker implantation for brady-tachy syndrome 14 and 20 months after STAR. – Three patients died during the FU: one after transcatheter aortic valve implantation 29 months after STAR, one while sleeping at the age of 93 (35 months after STAR), and one as a result of a road traffic accident (38 months after STAR). – One patient had an acute coronary ischaemic event following electrical cardioversion for symptomatic recurrence of AF (5 months after STAR).	AF recurrences and quality of life: – At a median FU of 42 months, all patients had experienced at least one AF recurrence; however, all patients reported a reduction in symptomatic arrhythmic events and, furthermore, all patients reduced the dosage and number of antiarrhythmic drugs – The quality of life markedly improved during the first 12 months after STAR (48 ± 15 at enrollment vs. 75 ± 15 at 12 months; *P* < 0.001). Electroanatomic mapping after STAR: – 6 patients (33%) underwent electroanatomic mapping of left atrium after STAR. Effective electrical isolation of the PVs was documented in 5 patients. One patients had PV electrical reconnections. No PV stenosis or phrenic nerve injury was documented.

### Safety endpoint

After 48 h of STAR, no acute side effects were recorded, but one patient, as already reported in the previous article (2), developed torsade de pointes one hour after treatment and was successfully treated with electrical cardioversion. At 42-month FU, the patient remains in good condition with a clear improvement in quality of life (the ICD recorded ventricular ectopies and only one atypical atrial flutter episode without AF or ventricular tachycardia recurrences).

In terms of side effects, at a median FU of 42 months (range 29–53), no acute or late toxicities were reported, and no serious adverse events (grade 3 or higher) occurred. Mild cases of esophagitis, likely due to the close anatomical relationship between the esophagus and the PV, occurred soon after STAR treatment and resolved fully (2). No patient has reported any further episodes of epigastric pain during the FU period so far.

At 12-month FU, 8 patients (44%) developed an asymptomatic, mild pericardial effusion (≤2 mm); at long term FU, only one patient still has a minimal, asymptomatic effusion, while the others have fully resolved.

A patient with known calcific degeneration of the aortic valve (mild stenosis before STAR treatment) developed progressive valve degeneration with severe stenosis documented. No other significant changes were observed on echocardiograms performed during FU visits. Regarding the left atrial strain (LAS) reservoir, at 6 and 12 months after STAR, it was lower in patients with AF episodes than in those without (7).

Two patients (11%) underwent pacemaker implantation for brady-tachy syndrome 14 and 20 months after radiotherapy.

Three patients (16%) died during the FU: one after undergoing transcatheter aortic valve implantation at another hospital (not related to STAR), 29 months after STAR, one while sleeping at the age of 93 (35 months after STAR), and one as a result of a road traffic accident, 38 months after STAR.

One patient (5%) discontinued FU at 6 months, refusing further control visits after experiencing an acute coronary ischaemic event following electrical cardioversion for symptomatic recurrence of AF (5 months after STAR). Nevertheless, the patient was subsequently contacted by telephone and reported a significant reduction in both the frequency and severity of AF episodes.

### Efficacy endpoint

Regarding efficacy, a 1-week ECG Holter monitoring performed 1 month after the procedure documented frequent symptomatic atrial ectopic beats and atrial tachycardias, but no AF recurrences. In most cases, during long-term FU, patients themselves initiated contact with our team to report arrhythmic recurrences, allowing us to observe the clinical course and recurrence patterns in a real-world setting.

At a median FU of 42 months, all patients had experienced at least one AF recurrence; however, all patients reported a reduction in symptomatic arrhythmic events and, furthermore, all patients reduced the dosage and number of antiarrhythmic drugs. However, as many AF episodes in elderly patients are asymptomatic, our data may underestimate the true arrhythmia burden.

All patients with recurrent arrhythmias were offered an electrophysiological study, which 6 (33%) accepted. In 5 patients the electroanatomic mapping of the left atrium showed the effective pulmonary vein isolation (PVI) (more specified data included in the previous paper) (2). Atrial mapping was performed using the CARTO System and Pentaray or Octaray mapping catheters (Biosense Webster, CA, USA), and no PV stenosis or phrenic nerve injury was documented.

**Figure 1:**
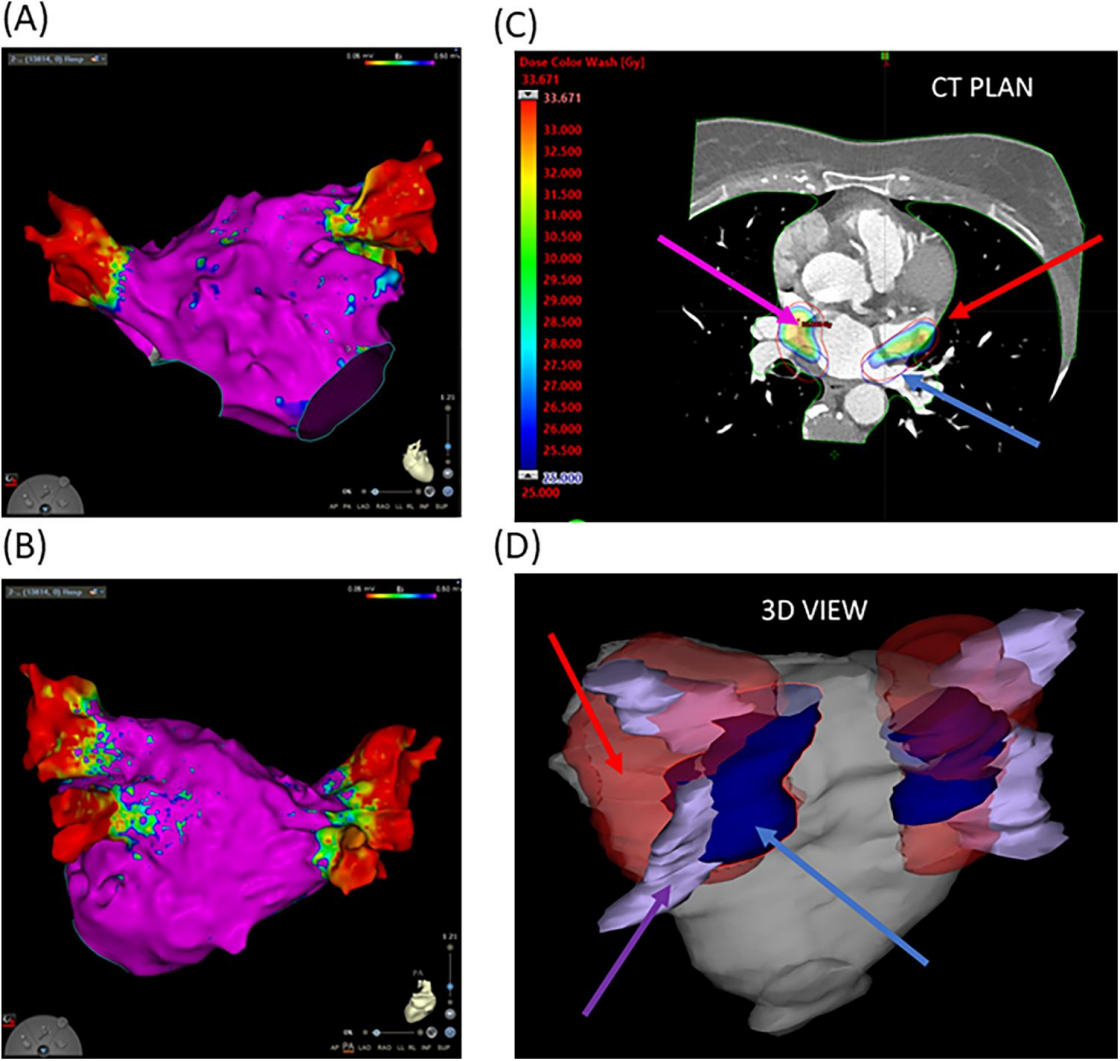
On the left **(A)** and **(B)**, electroanatomical mapping of the left atrium using the CARTO system and octaray catheter. Pulmonary veins were not isolated. On the right **(C)**, a treatment plan: the 25 Gy isodose is indicated by the red and pink arrows; the recurrence areas are in blue. **(D)** shows a 3D view: the planning target volume (PTV) is shown in red, the recurrence area in blue, and the pulmonary veins in purple. In the CT PLAN, the recurrence area was in a region where the STAR dose was less than 25 Gy.

The sixth patient performed electroanatomic mapping for atrial tachycardia episodes at 45 months after STAR. Electroanatomic mapping revealed abnormal potentials within the PVs. A careful analysis of the treatment plan showed that, to reduce radiation exposure to the esophagus, the lesion at the PV ostium received less than 25 Gy and therefore could not achieve a durable pulmonary vein isolation (Figure 1). This data confirms the main limitation of the treatment: the excessive anatomical proximity of at-risk structures, such as the esophagus and bronchi, could reduce the effectiveness of STAR.

Patient number 1, due to intestinal bleeding during oral anticoagulant therapy, underwent percutaneous closure of the left atrial appendage 4 years and 6 months after STAR. During this procedure, we documented the persistence of pulmonary vein isolation (PVI) using high-density electro-anatomical mapping. The patient had already undergone a previous mapping six months after STAR, documenting PVI (2).

EQ visual analogue scale (VAS) was used to assess quality of life. The EQ VAS records the patient's self-rated health on a vertical visual analogue scale from 0 (the worst health you can imagine) to 100 (the best health you can imagine) (2). Quality of life markedly improved during follow-up, potentially due to a reduction in symptomatic arrhythmic episodes (48 ± 15 at enrolment vs. 75 ± 15 at 12 months; *P* < 0.001). The quality-of-life assessment was not extended beyond 12 months because most patients, being elderly, developed multiple comorbidities over time, which would have significantly confounded the evaluation due to age-related conditions such as joint pain and other chronic disorders.

## Discussion

The STAR study was born from the desire to obtain PVI using a non-invasive method, suitable for older patients or those who cannot have or not desire an invasive procedure. The present phase-II LINAC-STAR trial is the first worldwide study designed to evaluate the safety of this treatment.

In terms of safety endpoint, the long-term adverse events with a possible correlation to STAR were mild esophagitis and mild pericardial effusion. Esophagitis is essentially due to the anatomical proximity of the esophagus and the PVs and we documented mild esophagitis without the onset of fistulas in a longer follow-up. We performed a “simultaneous integrated protection” dose for esophagus and bronchus, for reducing the risk of side effect. Moreover, STAR delivering treatment time was very short (3 min instead of 45–90 min for other technologies), reducing the risk of esophagus displacement during a longer treatment (2).

Regarding mild pericardial effusion, at long term FU, only one patient still had a minimal, asymptomatic effusion, while the others have fully resolved. It is necessary to monitor these events over a long time for potential development of chronic pericarditis.

For the “torsade de pointes”, as reported in the previous article (2), it is not possible to determine if this event was due to STAR or to a chance because in the patient's clinical history there are factors that can be associated with this type of event. However, at long term FU, the patient remains in good condition with a clear improvement in quality of life without AF or ventricular tachycardia recurrences). The other reported events do not appear to be related to STAR.

Regarding efficacy, all patients had experienced at least one AF recurrence; however, all patients reported a reduction in symptomatic arrhythmic events and, furthermore, all patients reduced the dosage and number of antiarrhythmic drugs. The main limitation of the efficacy endpoint is the lack of data regarding arrhythmic burden, especially since AF episodes can be asymptomatic in the elderly. Future studies will be necessary to analyze arrhythmic burden and compare STAR with catheter ablation. However, we have demonstrated for the first time that STAR is effective in performing PVI.

Only one patient had PV electrical reconnection confirming that the patient's anatomy is essential for structuring an optimal treatment plan and some unfavourable anatomies (mainly esophagus, PVs and left bronchus very close together) may not allow it.

In conclusion, the current data demonstrate the feasibility of STAR in elderly patients with paroxysmal AF and its potential to improve quality of life. The treatment remains experimental, and more comprehensive safety data are necessary; additionally, its efficacy will require further evaluation in a comparative study vs. catheter ablation.

## Data Availability

The original contributions presented in the study are included in the article/Supplementary Material, further inquiries can be directed to the corresponding author.
